# Effects of lexical skills and orthographic neighborhood size in word memory

**DOI:** 10.3758/s13421-023-01487-3

**Published:** 2023-11-09

**Authors:** Claire Ballot, Christelle Robert, Emilie Dujardin, Stéphanie Mathey

**Affiliations:** 1 University of Bordeaux, Labpsy, France; 2 Faculty of Psychology and Educational Science, University of Geneva, FPSE, Boulevard du Pont-d’Arve 40, CH-1211 Genève 4, Switzerland; 3 University of Poitiers, CNRS, CERCA, Poitiers, France

**Keywords:** Lexical skills, Orthographic neighborhood, Free recall, Memory recognition

## Abstract

Two experiments were designed to investigate the relationship between individual lexical skills in young adults and memory performance on words varying by their orthographic neighborhood size. In Experiment 1, a sample of 100 university students were administrated a set of spelling, reading, and vocabulary tests to assess their lexical skills. Then, they had to learn mixed lists of words from high and low neighborhood size and perform free recall and memory recognition tasks. Importantly, high lexical skills were found to enhance free recall and, to a lesser extent, recognition. In addition, a typical mirror effect of neighborhood size was found in recognition as words were better recognized and also produced less false alarms when they had a low neighborhood size. In Experiment 2, pure lists of words were designed and a new sample of 90 university students was assessed. We replicated the effect of lexical skills in free recall and the effect of neighborhood size for hits in recognition. Spelling skills were found to interact with neighborhood size in free recall in that low spelling skills were associated with a facilitatory effect of neighborhood size. In recognition, a relation between reading skills and neighborhood size was found such that the higher the reading skills, the higher was the inhibitory effect of neighborhood size. These results provide new evidence of an influence of lexical skills in word memory performance and underline the role of orthographic neighborhood size in episodic memory tasks.

## Introduction

These past decades, several studies have shown that lexical characteristics of to-be-learnt words influence memory recall and recognition (see, e.g., Cortese et al., 2004; Lau et al., 2018). Among these characteristics, the set of words that are orthographically similar, referred to as orthographic neighborhood, was found to affect episodic memory performance (e.g., Ballot et al., 2021; Cortese et al., 2004; Glanc & Greene, 2012). Apart from word characteristics, individual characteristics constitute another potential source of variation of episodic memory performance. Given the increase in memory complaints with aging (e.g., Balota et al., 2000), many studies have focused on individual age-related differences in memory performance (e.g., Shing et al., 2010; Smith, 2006). In comparison, the effects of individual differences in healthy young adults in word memory performance have been much less investigated (see Kirchhoff, 2009, for a review on individual differences in self-initiated encoding strategy use). Lexical skills are an individual characteristic that deserves to be investigated in young adults who perform memory tasks with words. There are significant individual differences in lexical skills among university students who are usually considered as skilled readers, and these differences affect visual word identification (e.g., Andrews, 2012, 2015; Dujardin et al., 2022). Whether such individual lexical skills also influence word memory performance in young adults, in addition to word characteristics already known to play a role in memory, remains to be established. The present study therefore investigated the role that differences in individual lexical skills and orthographic neighborhood size of the words to be learnt play in word memory in a population of young adults.

The use of verbal material in memory tasks raises the question of the putative role of individual language abilities in word memory performance. Differences in lexical skills assessed by using tests measuring spelling, reading, and vocabulary have even been observed in higher-education students (see, e.g., Andrews, 2015; Dujardin et al., 2022; Perfetti, 2007). Such differences in lexical skills could be underpinned by the lexical quality of word representations (i.e., the precision and flexibility of lexical knowledge that vary across individuals; Andrews, 2015; Perfetti, 2007). Adults with high lexical quality would thus have better reading comprehension, confuse meanings less, learn new words more efficiently, and have more stable orthographic representations (Perfetti, 2007). Such differences in lexical skills were found to influence visual word recognition (Andrews & Hersch, 2010; Andrews & Lo, 2012; Dujardin & Mathey, 2020). More precisely, high lexical skills have been shown to promote the speed and/or accuracy of responses in several word recognition tasks such as lexical decision, progressive demasking, naming (Dujardin & Mathey, 2020, 2022), and masked priming (Andrews & Hersch, 2010; Andrews & Lo, 2012). According to the lexical quality hypothesis (Perfetti, 2007), when a word is presented, individuals who benefit from more accurate lexical representations activate the lexical representation of that word more easily, which speeds up and increases the accuracy of lexical access. In line with this assumption, lexical skills were also found to interact with the word’s orthographic neighborhood, although this interaction was shown to be task-specific as it was observed in masked priming (Andrews & Lo, 2012) and naming tasks (Dujardin & Mathey, 2020, 2022) but not in standard lexical decision and progressive demasking tasks (Dujardin & Mathey, 2020). Regarding the link between lexical skills and memory performance, most evidence comes from developmental studies conducted in children. More precisely, lexical skills have a positive influence on children’s working memory performance (e.g., Gray et al., 2019; Masoura et al., 2021) as well as on children’s ability to recall events (e.g., Kulkofsky et al., 2008). Klemfuss (2015) further observed that relations between language skills and children’s recall vary by the type of language skill assessed and by the type of recall. In adults and as regards learning the meaning of rare unknown words, Perfetti et al. (2005) showed that skilled readers learned more new words than less skilled readers did. Event-related potentials measures suggested that learning produced a stronger memory trace of the word in skilled readers than in less skilled readers. The authors concluded that skilled readers are more able to use their word knowledge to add new words to their vocabulary, learn new verbal associations, or remember specific episodic information. In sum, the lexical quality of lexical representations seems to play a critical role in word processing and in the way, words are stored in memory.

Previous studies have found evidence of an influence of orthographic neighborhood size on memory performance (e.g., Ballot et al., 2021; Cortese et al., 2010, 2014; Justi & Jaeger, 2017). Orthographic neighborhood has also been considered as an index of orthographic distinctiveness (Glanc & Greene, 2012) and refers to the orthographic similarity between a given stimulus and other lexical representations (e.g., Chen & Mirman, 2012). Orthographic neighborhood size corresponds to the number of words sharing all but one letter in the same position with the stimulus (Coltheart et al., 1977): For example, the word *sleet* has seven neighbors (*N* = 7, i.e., *sleep, fleet, sheet, skeet, sweet, slept, sleek*). A mirror effect of *N* has been repeatedly found in recognition memory: The number of correctly recognized words is usually higher while the false-alarm rate is lower in low-*N* words than in high-*N* words (Ballot et al., 2021; Cortese et al., 2004, 2010, 2014; Glanc & Greene, 2007, 2012). Recent studies have shown that orthographic word features are particularly associated with better memory recognition performance than free recall, suggesting that these word properties play a more important role in tasks that may be driven by a memory process of familiarity (Ballot et al., 2021; Lau et al., 2018). At a theoretical level, several word recognition models (e.g., McClelland & Rumelhart, 1981, for visual word recognition; Luce & Pisoni, 1998, for spoken word recognition) posit that (1) the process of word identification involves discriminating among lexical items in memory that are activated on the basis of stimulus input, and (2) discrimination is a function of the nature (i.e., lexical similarity and frequency) of lexical items activated by the stimulus input. Some authors have proposed to extend the principles of interactive activation and competition models of visual word identification to account for the effects of orthographic neighborhood in the field of word memory (e.g., Chen & Mirman, 2012; Cortese et al., 2004). Within this framework, orthographic neighbors become activated upon the presentation of the stimulus word and interfere during the encoding of the to-be-learnt word. Consequently, high-*N* words are remembered less well than low-*N* words. The question then arises as to whether the effect of orthographic neighborhood size found in memory performance is sensitive to individual lexical skills, since a link between the effect of orthographic neighborhood and lexical skills has already been found in visual word identification (see Andrews, 2015; Dujardin & Mathey, 2020).

The overall goal of the present study was to investigate whether and to what extent the lexical skills of young adults influence performance across episodic memory tasks for words varying in orthographic neighborhood size. The effects of spelling, reading and vocabulary skills (see Andrews & Lo, 2012; Dujardin et al., 2022) were investigated in free recall and in memory recognition tasks. Based on the lexical quality hypothesis (Perfetti, 2007), higher lexical skills should be associated with higher word memory performance. In line with previous studies, we also expected an effect of orthographic neighborhood size (e.g., Ballot et al., 2021; Cortese et al., 2004), particularly in memory recognition. Finally, based on previous data in the field of visual word recognition (e.g., Andrews & Lo, 2012; Dujardin & Mathey, 2020), a relationship between individual lexical skills and the effect of word orthographic neighborhood could also be observed in memory performance. List composition (i.e., mixed vs. pure word learning lists) was changed across two experiments since previous studies suggested an influence of the context of encoding in the lexical effects observed in episodic memory tasks (e.g., Hunt & Eliott, 1980).

## Experiment 1

In the first experiment, we investigated the effects of both the lexical skills of participants and the orthographic neighborhood of words in free recall and memory recognition using a mixed-list design. Lexical skills were measured by evaluating the spelling, reading, and vocabulary skills of young adults across six language tests (e.g., Andrews & Lo, 2012; Dujardin & Mathey, 2020). The neighborhood size of the words to be memorized was manipulated by considering the number of orthographic neighbors (see also Ballot et al., 2021; Cortese et al., 2004). A classical mixed list design was used so that words from both neighborhood conditions were mixed across learning lists (e.g., Ballot et al., 2021; Glanc & Greene, 2007, 2012).

### Method

#### Participants

For an expected medium effect size (see also Hersch & Andrews, 2012), we estimated that a sample size of 85 participants was necessary to detect an interaction effect with a statistical power of .80. By the recruitment procedure, we came up with 100 adults aged 18 to 33 (*M* = 21.36 years; *SD* = 2.62; 73 women, 27 men). They were all native French speakers, reported normal or corrected-to-normal vision, and no language impairment. They had an average level of education of 14.55 years (*SD* = 1.34). A set of six tests including reading, spelling and vocabulary measures for the French language was administrated to assess individual lexical skills (see Dujardin & Mathey, 2020, 2022).

##### Reading skills

Participants had to read two texts aloud. The first test, Alouette-R (Lefavrais, 2005), consisted of a text with low-frequency words whose combination did not make sense. Participants had to read the whole text within 3 minutes. A reading speed score was calculated. The second test was “Le Pollueur” from test battery ECLA-16+ (Gola-Asmussen et al., 2011). Participants were instructed to read the text as quickly and accurately as possible for one minute. The number of correctly read words was recorded.

##### Spelling skills

Two tests taken from ECLA-16+ (Gola-Asmussen et al., 2011) were used to measure spelling skills. In the first test, a short text consisting of four sentences was dictated. Among the 83 words composing the text, the spelling of 10 target words related to usage spelling and 10 words related to agreement spelling was measured. The number of correct responses was recorded (score out of 20). For the second test, 10 irregular words, 10 regular words and 10 pseudowords were dictated. Accuracy associated with each list was recorded (score out of 10 for each list).

##### Vocabulary skills

Two vocabulary tests were used. First, the Mill Hill vocabulary test (Deltour, 1998) consisted in determining, for 34 words, the synonym corresponding to each word among six proposed alternatives. The number of correct answers was recorded (score out of 44). The second test was the LexTALE-FR vocabulary test (Brysbaert, 2013), in which participants were instructed to select the French words in a list of 84 sequences of letters. The Ghent score was measured from the number of correctly selected words and the number of nonselected nonwords.

All scores were then transformed into standardized scores and averaged to compute a general composite score corresponding to an average measure of lexical skills. Moreover, we derived a spelling skill score by averaging the regular word (*M* = 8.84, *SD* = 1.16), irregular word (*M* = 7.13, *SD* = 1.87), pseudoword (*M* = 9.24, *SD* = .76), and text dictation scores (*M* = 17.8, *SD* = 1.57). A reading score was obtained by averaging the Alouette-R reading speed scores (*M* = 554.72, *SD* = 94.64) and the reading accuracy scores from the Pollueur (*M* = 197.61, *SD* = 25.83). Finally, a vocabulary skills score was computed by averaging the Ghent (*M* = 44.65, *SD* = 5.70) and the Mill Hill scores (*M* = 34.37, *SD* = 4.34).

#### Materials

A total of 48 words with 4–6 letters and 1–2 syllables were selected from Lexique 3.8 (New et al., 2007). Two orthographic neighborhood size conditions were set up: 24 words had a high *N* (greater than or equal to six orthographic neighbors, e.g., *douche[shower]/couche[diaper], louche[ladle], mouche [fly], souche[strain], touche[touch], bouche[mouth]*) and 24 other words had a low *N* (lower than or equal to three orthographic neighbors, e.g., *dinde[turkey]/diode[diode]*)*.* Word imageability and subjective word frequency ratings were selected in the lexical database of Desrochers and Thompson (2009). The two word conditions were matched on the number of letters, number of syllables, objective and subjective frequencies and word imageability (*p*s >.10). The main statistical characteristics of the materials are presented in Table 1. In the free recall task, four mixed lists of 12 words each were constructed. Each list consisted of six words with a high *N* and six words with a low *N*. In the memory recognition task, 48 new words with the same characteristics as the learning words were selected. The list of stimuli is available in the Appendix Table 2.

**Table 1:** Characteristics of the word materials

Variable	High *N*	Low *N*	*p* value
*N*	8.41	1.12	<.001
OLD20	1.39	1.84	<.001
Number of letters	5	5	–
Number of syllables	1.33	1.33	–
Word frequency	7.49	7.14	.90
Subjective frequency	3.36	3.24	.60
Imageability	4.84	4.86	.90

*N* = number of orthographic neighbors. OLD20 = Orthographic Levenshtein Distance (Yarkoni et al., 2008). Word frequency taken in Lexique 3.8 (New et al., 2007) is given in number of occurrences per million. Scores of subjective frequency and imageability ranged from 0 to 7 (Desrochers & Thompson, 2009)

#### Procedure

After having signed a written informed consent form, participants performed a free-recall task computed with E-Prime 2.0 software. During the study phase, they had to memorize a list of words presented one by one. Each word appeared in lowercase at the center of a computer screen for 3,000 ms and was preceded by a 1,000-ms fixation cross. At the end of the list, a screen instruction asked each participant to count down for 30 s. Next, participants had to write the words they remembered on a sheet of paper. No time limit or order constraints were set for recall. This procedure was reproduced for all four lists of words. The presentation of the words was randomized within each list and the order of the list was counterbalanced across participants to control for list order effects. Following the four study and recall phases, they performed a recognition task in which 96 words were presented on the computer screen in a different random order for each participant. The 48 words from the previous recall task were mixed with 48 new words used as distractors. Participants were asked to decide whether each word appearing on the screen was “new” or “old” by pressing one of two buttons on the computer keyboard. The buttons were tailored to the participant’s laterality so that they would respond “old” with the dominant hand and “new” with the other hand. The words were preceded by a 1,000-ms fixation cross and remained on the screen until the participant responded. Following the memory tasks, participants completed the spelling, reading and vocabulary tests.

### Results and discussion

Generalized linear Mixed-model analyses were performed separately on the hits for recall, hits for recognition and false alarms as a function of orthographic neighborhood size (high vs. low) and the lexical skills (considered as a continuous factor) as fixed effects. Individual general lexical skills were first estimated with a general composite score calculated by averaging the scores for all the tests completed by the participants. Reading, spelling, and vocabulary skills were then considered separately for each participant by averaging the scores for the tests measuring each of these three skills. Random effects were estimated for each analysis using the method proposed by Baayen et al. (2008) based on a model comparison approach to determine random effects for each analysis (see also Freeman et al., 2010). For each analysis, the random intercept and the random slopes for both participants and items were estimated and compared. The analysis for participants and items as random intercept has been chosen as models, and exceptions are expressed in footnotes.

#### Free recall task

##### Analysis as a function of the general composite score^1^

**Fig. 1:**
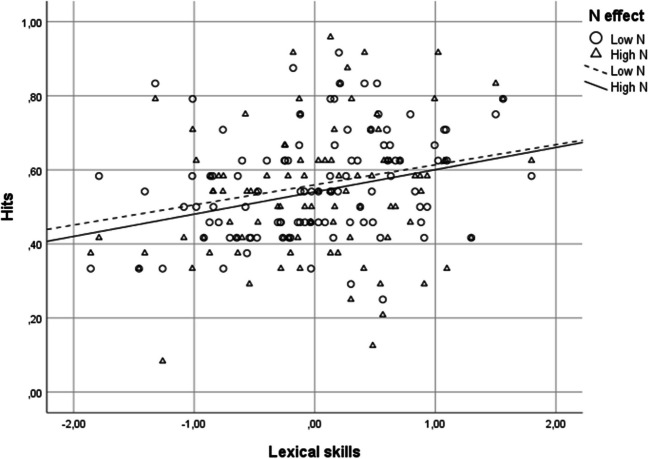
Mean hit rates for recall as a function of lexical skills and orthographic neighborhood in Experiment 1

Results are shown in Fig. 1. The analyses of hits showed that the general composite score predicted the percentage of correctly recalled words significantly and positively, *b* = .24, *z* = 2.76, *p* = .006, CI [.07, .41], *OR* = 1.28. The higher the general composite score of young adults, the more words they recalled correctly. The main effect of neighborhood size was not significant, *z* < 1, nor was the interaction with the general composite score, *z* < 1.

##### Analysis as a function of spelling, reading and vocabulary scores

Vocabulary scores tended to predict hits, *b* = .18, *z* = 1.78, *p* = .07, CI [−.018, .37], *OR* = 1.20. The higher the vocabulary scores of young adults, the more words they tend to correctly recall. Spelling scores tended to predict word recall, *b* = .15, *z* = 1.64, *p* = .09, CI [−.03, .34], *OR* = 1.17. The higher the spelling scores of young adults, the more words they tended to recall. Finally, the reading level did not significantly predict the hits for recall. The main effect of *N* was not significant, nor was the interaction between *N* and the three different scores (reading, spelling, and vocabulary), *z*s < 1.

#### Recognition memory task

##### Hit analysis as a function of the general composite score

The general composite score did not significantly predict the hits for recognition, *z* = 1.56, *p* = .12. The *N* effect was significant, *b* = −.48, *z* = −2.6, *p* = .009, CI [−.85, −.12], *OR* = 1.22, low-*N* words being better recognized (*M* = .87) than high-*N* ones (*M* = .82). The interaction effect between language skills and neighborhood size was not significant, *z* < 1.

##### Hit analysis as a function of spelling, reading, and vocabulary scores^2^

First, vocabulary scores significantly predicted the hits for recognition, *b* = .28, *z* = 1.99, *p* = .047, CI [.004, .55], *OR* = 1.32. The higher the vocabulary scores of younger adults, the more words they recognized correctly. Reading and spelling scores did not predict the hits for recognition, *z*s < 1. The *N* effect was significant, *b* = −.51*, z* = −2.61, *p* = .009, CI [−.88, −.12], *OR* = .60, indicating that low-*N* words were better recognized (*M* = .87) than high-*N* ones (*M* = .82). The interaction between *N* and vocabulary scores was not significant nor were the interactions between *N*, spelling level and reading scores, *z*s < 1.

##### False-alarm analysis as a function of general composite score

**Fig. 2:**
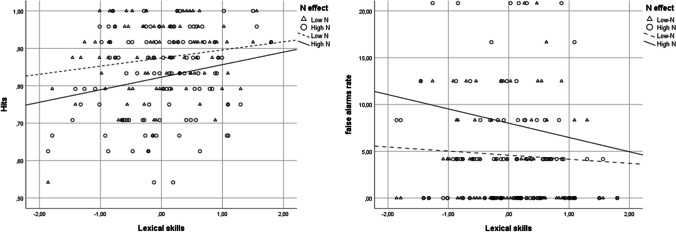
Mean hit (on the left) and false-alarm (on the right) rates for recognition as a function of lexical skills and orthographic neighborhood in Experiment 1

Results are shown in Fig. 2. The general composite score did not significantly predict false-alarm rates, *z* < 1. The main *N* effect was significant, *b* = .65, *z* = 2.25, *p* = .02, CI [.08, 1.22], *OR* = 1.93, with high-*N* words producing more false alarms (*M* = .08) than low-N ones (*M* = .05). The interaction between N and the general composite score was not significant, *z* < 1.

##### False-alarm analysis as a function of spelling, reading, and vocabulary scores

The spelling scores did not predict false alarm rates, z < 1, nor did vocabulary skills, *z* = −1.17, *p* =.24, and reading scores, *z* = 1.03, *p* = .30*.* The *N* effect was marginally significant, *b* = .57, *z* = 1.94, *p* = .052, CI [−.005, 1.14], *OR* = 1.77. High-*N* words tended to produce more false alarms (*M* = .08) than low-*N* words (*M* = .05). Finally, the interaction between vocabulary scores and *N* was significant, *b* = −.53, *z* = −2.98, *p* = .002, CI [−.88, −.18], *OR* = .58. The lower the vocabulary skills, the higher the facilitatory *N* effect. The interaction between *N* and reading scores was not significant, *z* = 1.54, *p* = .12 nor was the interaction between spelling scores and *N*, *z* < 1.

The main finding of Experiment 1 was that individual lexical skills influence episodic memory performance. Higher lexical skills lead to higher hit rates in free recall. Decomposing the composite score showed that vocabulary score was linked to episodic memory performance, especially in recognition. As posited by Perfetti (2007), individuals with high lexical skills should benefit from more accurate lexical representations stored in their memory, thus facilitating the retrieval of learnt words and leading to better memory performance. One may also argue that individuals with higher lexical skills are more able to remember specific episodic information (see also Perfetti et al., 2005). Furthermore, the typical mirror effect of orthographic neighborhood was found in the recognition task (see also Ballot et al., 2021; Cortese et al., 2004; Glanc & Greene, 2009, 2012). Low-*N* words were better discriminated as they were better recognized and produced fewer false alarms than high-*N* words. These findings are consistent with previous data and provide further evidence that lexical neighbors are co-activated during the presentation of the to-be-learnt word, thus interfering with encoding (see Cortese et al., 2004). Importantly, in line with previous studies, the effect of orthographic neighborhood was observed in recognition but not in the recall task, suggesting that orthographic features play a greater role in recognition than in free recall (see Lau et al., 2018). Finally, the orthographic neighborhood of words was not found to interact with the individual lexical skills in memory performance. Since mixed lists were used in Experiment 1, it may be argued that the present findings are underpinned by list-specific mechanisms, as mixed lists are known to involve specific attentional processes during word encoding (e.g., Dewhurst & Parry, 2000; Hunt & Eliott, 1980). Experiment 2 addressed this issue by using a pure list design.

## Experiment 2

In Experiment 2, we made the same comparisons as those in Experiment 1. However, the two types of stimuli were blocked by list: Participants had to learn pure lists of high-*N* words and pure lists of low-*N* words. This manipulation allowed us to evaluate whether the effects of lexical skills and orthographic neighborhood found in Experiment 1 are list specific or more general (see also Cortese et al., 2004, for the same procedure). If they turn out to be general, the list-type change should therefore not produce any modification in the pattern of effects found in Experiment 1 (see also Cortese et al., 2004).

### Method

#### Participants

As in Experiment 1, we estimated that a sample size of 85 participants was necessary to detect the interaction effect with a statistical power of .80. By the recruitment procedure, we came up with 90 young adults aged 18 to 28 years (*M* = 20.38 years, *SD* = 2.49; 69 women and 21 men) who had not participated in Experiment 1. They all were native French speakers (or had learned French in preparatory school), reported normal or corrected vision, and no language impairment. They had an average level of education of 14.44 years (*SD* = 1.29). As in Experiment 1, we transformed the raw scores of each lexical skills test and calculated scores for each of the skills considered (average, spelling, reading, and vocabulary). The spelling skills score was calculated by averaging the regular word (*M* = 8.75, *SD* = 1.16), irregular word (*M* = 6.66, *SD* = 1.86), pseudoword (*M* = 9.11, *SD* = 1.19), and text dictation scores (*M* = 17.16, *SD* = 1.58). The reading score was obtained by averaging the Alouette-R reading speed scores (*M* = 527.25, *SD* = 91.94) and the reading accuracy scores from the Pollueur (*M* = 189.91, *SD* = 27.63). Finally, the vocabulary skills score was calculated by averaging the Ghent (*M* = 42.58, *SD* = 8.05) and the Mill Hill scores (*M* = 33.88, *SD* = 3.64).

#### Materials

The materials were the same as in Experiment 1. Four pure lists of 12 words each were designed. Each list consisted of either high-*N* words or low-*N* ones.

#### Procedure

The procedure was the same as in Experiment 1.

### Results and discussion

#### Free recall task

##### Analysis as a function of general composite score

**Fig. 3:**
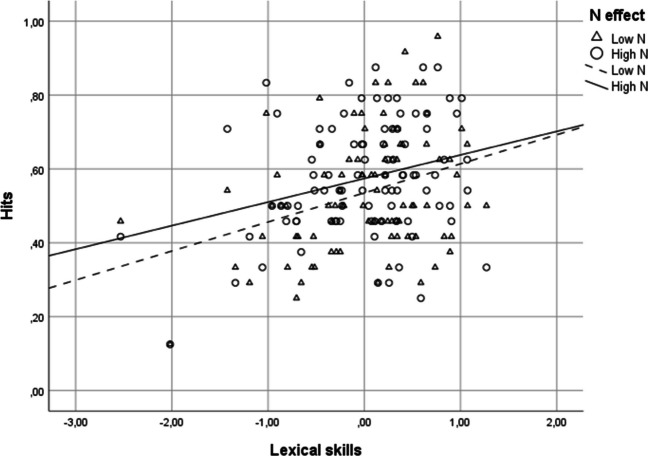
Mean hit rates for recall as a function of lexical skills and orthographic neighborhood in Experiment 2

Results are shown in Fig. 3. A significant main effect of general composite score was found, *b* = .36*, z* = 3.28*, p* = .001*,* CI [.14, .57], *OR* = 1.43*.* The higher the general composite score, the higher the number of hits. The *N* effect was not significant, *z* = 1.27, *p* = .20. The interaction between general composite score and *N* was not significant, *z* < 1.

##### Analysis as a function of spelling, reading, and vocabulary scores

Hits for recall were significantly predicted by spelling scores, *b* = .27, *z* = 2.65, *p* = .008, CI [.07, .48], *OR* = 1.32. The higher the spelling scores, the higher the number of hits. The reading scores did not predict hits, *z* = 1.48, *p* =.14. Finally, hits for recall were not predicted by vocabulary scores, *z* < 1. The effect of *N* was not significant, *z* = 1.27, *p* = .20. The interaction between *N* and spelling scores was significant, *b* = −.21, *z* = −2.32, *p* = .02, CI [−.39, −.03], *OR* = .81. The lower the spelling skills, the greater the facilitatory *N* effect. However, neither the interaction between *N* and reading scores, *z* = 1.45, *p* = .15, nor the interaction between *N* and vocabulary scores, *z* < 1, were significant.

#### Recognition memory task

##### Hit analysis as a function of general composite score

**Fig. 4:**
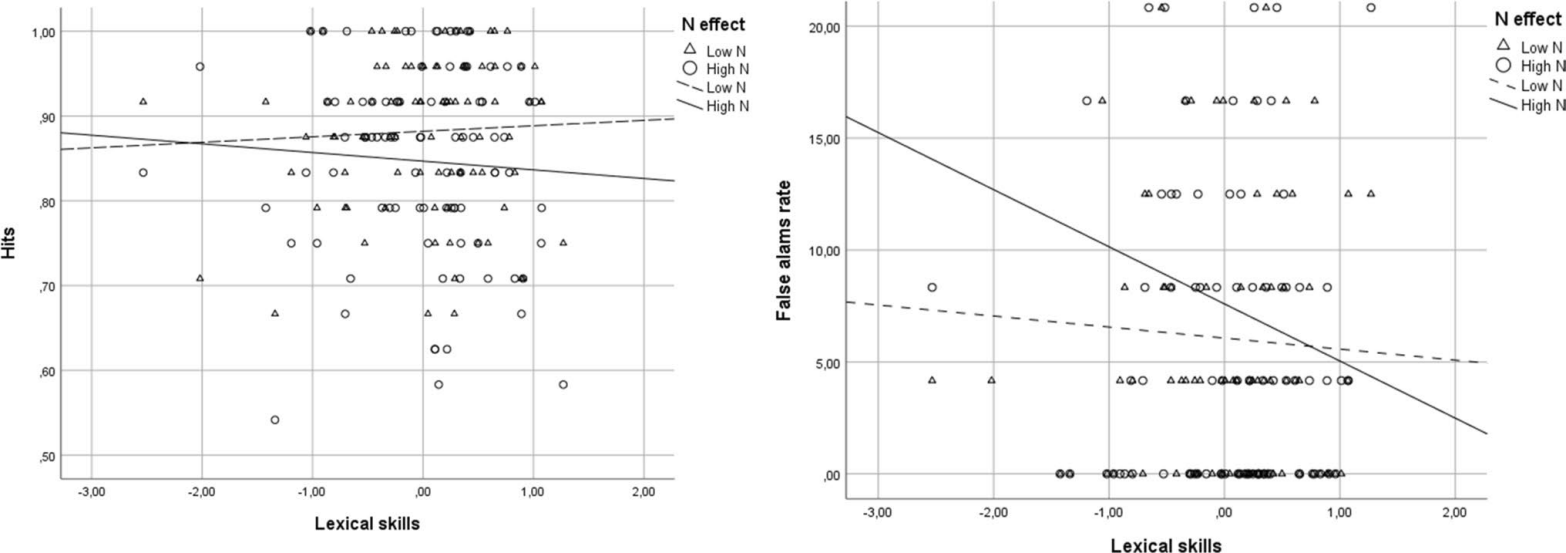
Mean hit (on the left) and false-alarm (on the right) rates for recognition as a function of lexical skills and orthographic neighborhood in Experiment 2

Results are shown in Fig. 4. No significant main effect of general composite score was found, *z* < 1. The main effect of *N* was significant, *b* = −.33*, z* = −2.01*, p* = .04*,* CI [−.65, −0.008], *OR* = .72. Low-*N* words were better recognized (*M* = .88) than high-*N* ones (*M* = .85). The interaction between general composite score and *N* was not significant, *z* = −1.10, *p* = .27.

##### Hit analysis as a function of spelling, reading, and vocabulary scores

Spelling and vocabulary did not significantly predict hits for recognition, *z*s < 1, nor reading scores, *z* = 1.08, *p* = .28. The effect of *N* was significant, *b* = −.33*, z* = −2.01, *p* = .04, CI [−.65, −.08], *OR* = .72. Low-*N* words were better recognized (*M* = .88) than high-*N* ones (*M* = .85). The interaction between *N* and reading scores was significant, *b* = −.23, *z* = −2.16, *p* = .03, CI [−.45, −.02], *OR* = .79. The higher the reading scores of individuals, the higher the inhibitory effect of *N* was. Finally, the interactions between N and vocabulary or spelling scores were not significant, *z*s < 1.

##### False-alarm analysis as a function of general composite score^3^

The main effect of general composite score was not significant on false-alarm rates, *z* = −1.06, *p* = .29. The *N* effect was not significant, *z* < 1. The interaction between N and general composite score was not significant, *z* = −1.47, *p = .*14.

##### False-alarm analysis as a function of spelling, reading, and vocabulary scores^3^

Spelling scores did not predict the false-alarms rates, *z* = −1.69, *p* = .10, nor did reading, *z* < 1 and vocabulary scores, *z* = 1.16, *p* = .24. The *N* effect was not significant, *z* < 1. The interaction between our two factors was not significant for vocabulary and spelling scores, *z*s < 1, while it was marginally significant for reading skills, *b* = −.15, *z* = −1.83, *p* = .07, CI [−.31, .01], *OR* = .86. The higher the reading skills, the lower the inhibitory *N* effect tended to be.

As in Experiment 1, we found an effect of lexical skills in free recall, as higher lexical skills were associated with a higher number of correctly recalled words. This finding provides further evidence that individuals with high lexical skills are more able to remember learnt words, probably owing to more accurate lexical representations stored in their memory (see Perfetti, 2007). By decomposing the general composite score, it appears that spelling skills were linked with recall performance. However, we did not observe any effect of lexical skills in recognition. Since free recall is considered to require more effort and to put a greater emphasis on retrieval mechanisms than recognition, it may be argued that the effect of lexical skills arises more in resource-demanding memory tasks and/or during memory retrieval. Individuals with more accurate word representations stored in their memory could thus retrieve these words more easily when performing memory tasks that require more self-initiation. Once again, we found an effect of orthographic neighborhood size in recognition, with low-*N* words being better recognized than high-*N* ones (see also Ballot et al., 2021; Cortese et al., 2004; Glanc & Greene, 2007, 2012). We therefore replicated the effect of orthographic neighborhood size in memory recognition for hits using a pure-list design, suggesting that this is a general effect unaffected by list composition during encoding. Also, we observed an interaction between neighborhood size and spelling skills in free recall. Individuals with the lowest spelling skills were those who exhibited the highest facilitatory effect of orthographic neighborhood size in free recall. It therefore seems that a word learning context that maximizes a relational processing between words during encoding (i.e., high-*N* words in pure lists; see also Saint-Aubin & Leblanc, 2005) favors individuals with low spelling skills. Finally, an interaction between *N* and reading skills was found in recognition. Individuals with the highest reading skills exhibited the strongest inhibitory effect of orthographic neighborhood size. It could be argued that when performing a memory task that requires reading skills (i.e., memory recognition), high reading skills favor an increase in the interference effect of orthographic neighbors during encoding and/or retrieval.

## General discussion

The most important finding of the present study is the clear evidence of the role of individual lexical skills in episodic memory performance as observed in university students, especially in free recall. Whatever the list composition (Experiments 1 and 2), higher lexical skills were associated with greater word recall. Second, the expected effect of orthographic neighborhood size was found especially in memory recognition, using both a mixed (Experiment 1) and a pure-list design (Experiment 2). Finally, specific lexical skills were found to modulate the effect of orthographic neighborhood size under a specific learning context (i.e., pure word list learning). These lexical skills differed depending on the task (i.e., spelling skills for recall, and reading skills for recognition). The results regarding the role of lexical skills differences in word memory and the role of orthographic word characteristics in episodic memory tasks are discussed below.

First, these results strongly suggest that word episodic memory performance is sensitive to the lexical skills of participants, especially in free recall and to a lesser extent in recognition. Within these lexical skills, spelling and vocabulary skills appeared to be more associated with memory performance than reading skills. In line with the lexical quality hypothesis (Perfetti, 2007), the present findings provide further evidence that individual differences in lexical skills lead to differences in the quality of lexical representations, hence modifying the performance that involves word retrieval. The present results extend data from visual word recognition (e.g., Andrews & Hersch, 2010; Andrews & Lo, 2012; Dujardin & Mathey, 2020) to the field of episodic memory. Since participants with higher lexical skills benefit from more accurate lexical representations in memory, encoding and retrieval mechanisms in memory tasks could be facilitated. Interestingly, the effect of lexical skills in recall was similar regardless of list composition, suggesting that it was not due to processes involving the other items in the list but rather to mechanisms associated with representations stored in memory (see also Cortese et al., 2004, for the same rationale). Finally, the effects of lexical skills were clearly found in free recall, regardless of list composition, while they only appeared for a specific lexical skill (i.e., vocabulary) and a specific context of encoding in recognition (i.e., mixed-list design). Given that recognition tasks are known to limit retrieval in memory by providing a cue (Lau et al., 2018), this finding suggests that lexical skills intervene particularly during recollection operations by facilitating the retrieval of words in memory. Note that, as in previous studies addressing the issue of lexical skills in visual word recognition (e.g., Andrews & Lo, 2012; Dujardin & Mathey, 2020; Andrews & Hersch, 2010), our participants were university students, resulting in lower variability in lexical skills than would be expected in a less educated adult population. Conducting this study on a sample with a lower educational level and/or a higher variability in lexical skills may presumably reveal more or higher effects of lexical skills in memory performance.

Second, orthographic neighborhood size was shown to influence word memory performance. The expected interference effect of orthographic neighborhood was consistently found in recognition (Experiments 1–2) but not in free recall, which is in line with previous evidence that orthographic word features play a lesser role in more demanding memory tasks such as free recall. As posited by Lau et al. (2018), this task-specific lexical effect could be because it is easier to establish a match between the provided cue in the recognition task and the word stored in memory. The effect of orthographic neighborhood found in recognition using a classical mixed-list design corresponded to a mirror effect. Low-*N* words were better recognized and produced fewer false alarms than high-*N* ones (Ballot et al., 2021; Glanc & Greene, 2007, 2012). Within an interaction activation and competition framework extended to memory performance (e.g., Chen & Mirman, 2012; Cortese et al., 2004), it may be argued that words that are orthographically similar to many other words receive interference from their neighbors that are coactivated during encoding. Owing to this interference, the memory trace is weakened. Importantly, the effect of orthographic neighborhood in correct recognition was observed regardless of list composition, suggesting that this effect is not list specific and is indeed triggered by the representations stored in memory (see also Cortese et al., 2004). A limitation of our study is that it does not allow us to distinguish between the effect of neighborhood size and that of neighborhood frequency since words with more neighbors have typically more higher frequency neighbors (e.g., Andrews, 1997), which was also the case here. However, it should be noted that even orthographic neighborhood frequency effects have been shown to influence visual word recognition (e.g., Grainger et al., 1989; Perea & Pollatsek, 1998; see Mathey, 2001, for a review), little evidence has been provided in memory (but see Roodenrys et al., 2002, for phonological neighborhood frequency effect in short-term memory). Justi and Jaeger (2017) have shown that neighborhood size plays a more important role than neighborhood frequency in free recall and memory recognition, while the reverse pattern is usually observed in the lexical decision task. Finally, it should be noted that we considered here the classical operationalization of orthographic neighborhood of Coltheart et al. (1977) in which letter position and length are held constant. We cannot exclude here that other kinds of orthographic neighbors, varying on these latter characteristics, might also have participated in our neighborhood effects. The influence of extended neighborhood, including neighbors with added or deleted letters have been reported in lexical access tasks (see, e.g., Yarkoni et al., 2008), suggesting that letter position has to be considered in models of word recognition in accordance with several proposals (e.g., overlap model, Gomez et al., 2008; SERIOL model, Whitney, 2001; SOLAR model, Davis, 2010; see also Grainger, 2008; Norris, 2013, for reviews). Although it was beyond the scope of our study to disentangle the effects of various orthographic neighbors in episodic memory, it can be noted that *N* and OLD 20 were logically varying in the same direction in our two conditions of orthographic neighborhood size so it is possible that extended neighbors have contributed to the neighborhood effect. Further studies should be designed to address specifically the role of such extended orthographic neighbors in memory performance.

Concerning the interaction between *N* and lexical skills, we found effects with specific skills depending on the memory task and learning list composition considered. In recognition, the effect of neighborhood size was found to interact with reading skills when pure lists were used only. Low-*N* words were better recognized than high-*N* ones as reading skills increased, suggesting that the interference of orthographic neighbors in memory recognition increases as reading skills increase. Under a pure-list learning context, reading skills seem therefore critical in performing a task that requires a decision on written words that have a number of orthographically similar neighbors. The combined effects of reading and *N* in recognition appears to be context dependent since it was here found to depend on the list composition in which it was presented, not just on the word per se. As proposed by Lau et al. (2018), such dissociations could reflect how the effects of word features depend on context parameters and how the memory system focuses on the word features that are most useful for optimizing performance depending on the task and instructions. In free recall, a puzzling finding was the facilitatory effect of orthographic neighborhood size associated with spelling skills observed in the pure-list design. In this specific context of encoding, lower spelling skills were associated with a better recall of high-*N* words as compared with low-*N* ones. Again, this effect seems to be context dependent. Pure lists are assumed to maximize the relational processing between words (e.g., Saint-Aubin & Leblanc, 2005), which could be more easily developed when words have a high neighborhood size (see Glanc & Greene, 2009) and when pure learning lists are used (see Saint-Aubin & Leblanc, 2005). Therefore, when encoding high-*N* words, participants with lower spelling skills could rely more on the orthographic similarities between words (which would be emphasized by their numerous neighbors) than on item processing (i.e., based on word features). This effect would particularly appear in tasks requiring spelling skills, such as free recall.

To conclude, we found evidence that both individual lexical skills of vocabulary, reading and spelling, and word orthographic neighborhood play a role in word episodic memory. These findings extend previous data collected in the field of visual word recognition (e.g., Andrews & Hersch, 2010; Andrews & Lo, 2012; Dujardin & Mathey, 2020), thus indicating that language skills also play a role in word memory. Moreover, the effect of orthographic neighborhood, observed particularly in memory recognition, provides further evidence that orthographic word features are mainly used in memory tasks driven by familiarity processes. The influence of orthographic neighborhood was found to be sensitive to individual lexical skills under specific experimental contexts (i.e., depending on list learning composition and memory task), suggesting that participants with low lexical skills (either reading or spelling skills depending on task constraints) are more sensitive to the influence of the orthographic neighborhood. Note that the recognition task was here always conducted after the free-recall task following the procedure used by Lohnas and Kahana (2013; see also Ballot et al., 2021) in order to allow comparisons with previous data from studies that only used a recall task (e.g., Cortese et al., 2004). However, we acknowledge that a limitation of such design is that prior word recall may have influenced recognition performance by enhancing encoding (see, e.g., Roediger & Karpicke, 2006). Future studies counterbalancing the order of recall and recognition tasks or using a single recognition task should be conducted to address this issue.

Finally, the results of this study highlight the importance of taking individual lexical skills into account as well as the orthographical features of the words when assessing episodic memory performance. Further studies should be designed to clarify the specific role of the various components of individual lexical skills in word memory across different learning contexts and memory tasks.

## Appendix

**Table 2:** List of stimuli

	**Low-** ***N***	**High-** ***N***
Learned words	octet[*byte*], scalp[*scalp*], geai[*jai*], fakir[*fakir*], lynx[*lynx*], dinde[*tukey*], orgue[*ogan*], mythe[*myth*], poivre[*pepper*], étui[*case*], globe[*globe*], joueur[*player*], argile [*clay*], balai[*broom*], galop[*gallop*], képi[*kepi*], stade[*stage*], grec[*greek*], volume[*volume*], cloche[*bell*], cerf[*deer*], plomb[*lead*], tronc[*trunk*]	volt[*volt*], cosse[*pod*], derme[*dermis*], soute[*bunker*], charte[*charter*], hotte[*hood*], maille[*mesh*], fard[*blush*], niche[*niche*], fente[*slope*], poire[*pear*], hache[*axe*], pape[*pope*], douche[*shower*], casque[*helmet*], four[*oven*], lama[*lama*], visée[*issue*], purée[*mash*], virage[*turn*], sapin[*fir*], menu[*menu*], paroi[*wall*], palier[*level*]
New words (distractors)	lion[*lion*]*,* jazz[*jazz*], frêne[*ash*], stèle[*stele*], flore[*flora*], noyau[*core*], clef[key], tricot[*knitting*], habit[*dress*], nylon[*nylon*], poulpe[*octopus*], timbre[*timbre*], studio[*studio*],moelle[*marrow*], chèvre[*goat*], épée[*sword*], fiord[*fjord*], musc[*musk*], recul[*hindsight*], vélo[*bike*], steak[*strak*], bonze[*bonze*], norme[*standard*], krill[*krill*]	aile[*wing*], scie[*saw*], gaine[*sheath*], vitre[*window*], loyer[*rent*], nain[*dwarf*], souris[*mouse*], muret[*low wall*], volet[*shutter*], craque[*crack*], hardes[*herds*], teinte[*colour*], palme[*palm*], clique[*clique*], ciment[*cement*], maïs[*corn*], figue[*fig*], vase[*vase*], débit[*debit*], juré[*juror*], verbe[*verb*], toque[*tick*], bulle[*bubble*], borne[*terminal*]

## Footnotes

1 For this analysis, 1|participants, 1|items and 1+neighborhood|participants were chosen as random effects.

2 For this analysis, 1|participants, 1|items, 1+neighborhood|participants and, 1+reading scores|items were chosen as random effects.

3 For these analysis, 1|participants, 1|item and 1+neighborhood|participants were chosen as random effects.
